# Editorial: Profiling immune responses to novel *Salmonella* vaccines: a path to clinical trials

**DOI:** 10.3389/fimmu.2026.1817651

**Published:** 2026-04-16

**Authors:** Giuseppe Stefanetti, Malick Mahdi Gibani, Fabio Fiorino

**Affiliations:** 1 Department of Biomolecular Sciences, University of Urbino Carlo Bo, Urbino, Italy; 2 Department of Infectious Disease, Faculty of Medicine, Imperial College London, London, United Kingdom; 3 Department of Medicine and Surgery, LUM University “Giuseppe Degennaro”, Bari, Italy

**Keywords:** immune profiling, Salmonella, vaccination, vaccine formulation strategies, vaccine candidates

Invasive and enteric diseases caused by *Salmonella* – including enteric fever due to *S.* Typhi and *S.* Paratyphi and invasive non-typhoidal *Salmonella* (iNTS) – remain major global health challenges. Antimicrobial resistance and the lack of licensed iNTS vaccines further underscore the need for candidates that are safe, immunogenic and well positioned for clinical translation. The life cycle of *Salmonella* includes extracellular and intracellular niches, suggesting that protection will require both humoral and cellular immunity. The relative contribution of different immune mechanisms at different stages of the life cycle is yet to be defined and there are currently no reliable immune correlates of protection suitable to support vaccine licensure.

This Research Topic was assembled to accelerate translational decision-making by focusing on immune profiling that moves beyond binding antibody titers alone. Across seven contributions, the Research Topic highlights immune quality and function (including mucosal immunity and bactericidal activity), outcome-linked cellular signatures in human settings and harmonized assays that enable comparability across studies. Collectively, these elements will help prioritize candidates for clinical development.

In their contribution, Haldar et al. report a candidate glycoconjugate strategy coupling an O-specific polysaccharide to a recombinant outer membrane protein carrier derived from typhoidal serovars, with the aim of achieving broader protection. The study integrates systemic and intestinal antibody readouts, functional measures and memory T-cell features, supporting the concept that breadth across serovars (including *S.* Typhimurium and *S.* Enteritidis) may be most credibly evaluated using multi-layered endpoints rather than single-parameter immunogenicity.

Improved vaccine formulation and inclusion of new adjuvants are recognized as pragmatic approaches to improve immunogenicity across target populations. Alugupalli explores a defined TLR4 ligand-based adjuvant formulation (“Turbo”) admixed with Vi polysaccharide vaccines, demonstrating improved anti-Vi IgG responses and improved bacterial control in mouse models, including infant animals. The broader implication is that controlled innate stimulation may help stabilize and amplify polysaccharide vaccine performance in age windows where responses are often suboptimal, an issue directly relevant to polysaccharide vaccines.

The Topic also includes work in a high-impact applied setting: control of *S.* Enteritidis in poultry. Suresh et al. evaluate mucosal adjuvants combined with an orally delivered chitosan-nanoparticle subunit platform, showing improvements in immune readouts and reductions in intestinal colonization after challenge. Beyond the immediate relevance to transmission control, the work reinforces that mucosal delivery, adjuvant choice and formulation can markedly shape protective phenotypes and should be assessed using endpoints linked to infection control, not serology alone.

Human correlates of protection remain a key translational bottleneck to vaccine development. Using a controlled human infection model, Booth et al. analyze circulating T follicular helper (cT_FH_) subsets following Ty21a vaccination and subsequent oral challenge with wild-type *S.* Typhi. Their findings link specific cT_FH_ subset patterns with protection following challenge, illustrating how integrated cellular–humoral profiling can reveal immune signatures that may guide candidate optimization.

Assay readiness and harmonization are frequently underappreciated requirements for clinical progression. These issues are addressed in two original research articles. Carducci et al. qualify a scalable anti-Vi IgG ELISA calibrated against international standards to enable cross-study comparability and they develop high-throughput tools to quantify both the magnitude and functional activity of antibodies against *S.* Paratyphi A. In areas where correlates are still emerging and vaccine pipelines are expanding, such standardized and functional assays provide enabling infrastructure—critical for consistent interpretation across sites and for evidence packages that support advancement.

Finally, McCann et al. review the role of controlled human infection models (CHIMs) as accelerators for invasive *Salmonella* vaccines, particularly for connecting intensive immune profiling to defined clinical endpoints in relatively small cohorts. The review also underscores the need to interpret CHIM findings in the broader context of endemic populations and to expand translational tools for *S.* Paratyphi A and iNTS vaccine development.

Taken together, the studies in this Research Topic highlight key priorities for the field, ranging from rational antigen design, breadth of immunity, improved formulation strategies that improve immunogenicity (including defined innate agonists and mucosal approaches), human immune profiling that links cellular and humoral features to outcome and harmonized functional assays that enable comparability across trials. Integrating these elements will help move from descriptive immunogenicity toward predictive immunology—supporting better-informed decisions as novel *Salmonella* vaccines progress toward clinical trials.

